# Moderating Role of Information Asymmetry Between Cognitive Biases and Investment Decisions: A Mediating Effect of Risk Perception

**DOI:** 10.3389/fpsyg.2022.828956

**Published:** 2022-03-22

**Authors:** Mingming Zhang, Mian Sajid Nazir, Rabia Farooqi, Muhammad Ishfaq

**Affiliations:** ^1^School of Business, Macau University of Science and Technology, Macau, Macao SAR, China; ^2^Institute of Administrative Sciences, University of the Punjab, Lahore, Pakistan; ^3^Department of Psychology, University of Central Punjab, Lahore, Pakistan; ^4^Faculty of Management Sciences, Riphah International University, Faisalabad, Pakistan; ^5^Department of Management Sciences, Comsats University Islamabad, Lahore, Pakistan

**Keywords:** optimism bias, anchoring bias, risk perception, information asymmetry, decision making

## Abstract

Behavioral Finance is an evolving field that studies how psychological factors affect decision making under uncertainty. This study seeks to find the influence of certain identified behavioral financial biases on the decision-making process of investors in developing countries. This research examines the moderating effect of Information asymmetry on the two most important and commonly used cognitive biases, namely Anchoring bias and Optimism bias and decision making and investigates whether Risk perception mediates the relationship between them or not. Quantitative research has been conducted using a structured questionnaire for data collection. After completing the pilot study, a questionnaire was designed and sent to investors *via* online channels. Data has been collected from 317 real estate investors. Mediation analysis has been performed using model 4 and moderation analysis by applying model 15 of Process Macros (Hayes, 2017) for the interaction effect. The study investigated that both cognitive biases have a significant positive effect on investors’ decisions and Risk perception also significantly mediates the relationship between them. Consistency with other studies suggests that Information asymmetry has a significant moderating effect. The proposed conceptual model provides insight into how investors’ decisions are influenced by behavioral biases in the real estate sector and enhances the understanding of cognitive biases in the real estate sector. This study is recommended for real estate investors and policymakers of emerging and developed countries. The current study is the first of its kind, focusing on cognitive biases on investment decisions with mediating role of Risk perception and the moderating effect of Information asymmetry.

## Introduction

Standard finance is also termed traditional finance based on diverse theories and principles. It has built upon the Portfolio theory (Rubinstein, 2002), Arbitrage Principles (Modigliani and Miller, 1958) and the Efficient Market Hypothesis (EMH) (Malkiel and Fama, 1970). These theories have common assumptions that the market is efficient, investors are rational, and their decisions are not affected by cognitive errors.

The rationality was first challenged by Simon (1956), who investigated that rationality is limited and affected by external and internal factors. After the energy crisis of the 1970s, behavioral finance transpires as a new concept with a combination of psychological aspects and financial decisions (Kahneman and Tversky, 1979), which established a milestone of individual behavior in finance. They argued that people prefer gains and losses differently as investors’ decisions are based on perceived gains instead of losses. Expected utility theory by Fishburn (1988) stated that people construct decisions through rational resources since they possess unlimited rationality and an approach to useful and perfect information that is why they can make rational decisions (Caplin and Leahy, 2001).

The paradigm of behavioral finance is shifted from traditional finance. The origin of behavioral finance took place by the idea of Tversky and Kahneman (1979), prospect theory, led by the expected utility theory. If investors know about the market information and behavior, their irrational behavior still exists because investors fear the future in terms of loss. According to the classical financial paradigm, researchers have focused on behavioral finance reactions regarding investors’ profit fluctuations in explaining the investor’s decisions. Behavioral finance focuses on different beliefs that encourage people to act unreasonably. Many people have different mental biases that may change into the biggest hurdle to grow their money or wealth.

The cognitive biases and real estate investment has scarcely been studied. Besides, none of the prior studies known to the researcher has been conducted in the context of developing countries. The real estate sector is integral component of the economy in all countries. Several technological developments have changed the economy and promoted growth throughout the world. The real estate sector and its business models face transformations caused by fundamental changes in technology, economy, and society (Pfnür and Wagner, 2020). Therefore, the complexity and diversity of real estate markets and their interdependence with the economy require further investigation (Pandey and Jessica, 2018). In addition, real estate investments are considered a complex human cognitive process in which decisions are made about potentially uncertain future returns. The real estate sector plays a vital role in ensuring a sustainable economy. Current political and economic matters, such as high inflation rates, cause real estate prices to rise sharply (Zain-ul-Abdin et al., 2019).

Anchoring is one of the most robust cognitive biases with implications in all decision-making processes (Furnham and Boo, 2011). When people rely on anchor value or explore anchor information, bias occurs (Jung and Young, 2019). In Anchoring bias, investors depend on a piece of initial information (Shin and Park, 2018). An investor starts with the initial approximation and then makes judgments based on additional information (Sharot, 2011).

Optimism bias enhances the positivity in investors’ minds, which is why they perceive the event more favorably (Anderson and Galinsky, 2006; Englmaier, 2010). In literature, Optimism bias is directly linked with the overreaction of information (Trevelyan, 2008). This overreaction enhances the investor’s confidence and becomes optimistic (Barros and Di Miceli da Silveira, 2007; Gudmundsson and Lechner, 2013).

Risk perception is the essential component of decision-making (Singh and Bhowal, 2010). Risk is a complex and essential factor in understanding and investigating. Speculators with an abnormal state of budgetary proficiency favor values while financial specialists with low money-related education lean toward bank stores. Risk perceptions are beliefs about potential harm/profit or the possibility of a loss. It is a subjective judgment that people make about the characteristics and severity. The degree of risk associated with a given behavior is generally considered to represent the likelihood and consequences of harmful effects that result from that behavior. Risk perception is a highly personal process of decision making, based on an individual’s frame of reference developed over a lifetime, among many other factors (Robinson and Marino, 2015).

The main objective of this study is to check the effects of cognitive biases on decision making and *via* the mediating role of Risk perception. It also strives to explore the moderating role of Information asymmetry for investors’ awareness about the hazards of Information asymmetry when making investment decisions.

## Theoretical Background

Modern finance has been dismissing the concept of the EMH introduced by Fama (1970), which prompts the advancement of behavioral finance because psychology is becoming deciphered as a key component (Chaffai and Medhioub, 2014). Rubinstein (2002) stated that people construct decisions through rational resources. Firstly, rationality was challenged by Simon (1956), who investigated that rationality is limited and also affected by some other external and internal factors.

### Prospect Theory

Within prospect theory, Kahneman and Tversky (1979) described that when the outcome of a decision is uncertain, an investor will focus on making a profit instead of reducing losses. This theory also states that when two choices are available in the securities market (in the shape of profit and losses), the investor’s preference is based on the perceived returns instead of losses. This theory is related to investors’ judgments called cognitive biases and their affect on investment decisions.

## Hypotheses Development

### Optimism Bias and Investment Decision Making

A huge literature underlined the numerous biases of investors’ behavior such as Optimism bias, Anchoring bias, availability bias, and overconfidence bias to explain economic decisions (Zacharakis and Shepherd, 2001). Investors believe in their mental conditions and shortcuts when making decisions (Maguire and Albright, 2005). Scheier and Carver (1985) have revealed optimism as derived positive beliefs and expectations about upcoming future incidents. If a person has a high level of optimism, he can expect higher subjective well-being in his good or bad times. The optimism comes face-to-face when he underestimates the negative happenings and will consist in anticipation of great results (Limongi et al., 2019). Another study also revealed that people with higher optimistic feelings are interested in more risky investments because they are not afraid of failing (Anderson and Galinsky, 2006).

From the above discussion, a hypothesis is generated:

**H1:** Optimism bias has a significant positive effect on investment decisions.

### Anchoring Bias and Investment Decision Making

People move to depend on their judgments when they just utilize the available information (Costa et al., 2017). Tversky and Kahneman primarily initiated this Anchoring bias in 1974. Anchoring bias is a cognitive bias that occurs when prices are fixed (anchored) by recent and contemporary observations. Investors mostly use the previous price as a reference for making the regulations (Kahneman and Riepe, 1998; Costa et al., 2017; Lieder et al., 2018). Meanwhile, Anchoring bias is a situational bias that varies as per the situation at the time of decision making (Kartini and Nahda, 2021). Anchoring bias happens when individuals depend on previous data they find when deciding (de Wilde et al., 2018; Lieder et al., 2018). The prior studies have explored that the Anchoring effect positively influences investors’ decision-making process (Chaudhary, 2013). An empirical study investigated that Anchoring bias significantly affects financial decision-making (Costa et al., 2017) and most commonly used bias in managerial and investment matters (Gaudet et al., 1998; Shin and Park, 2018). In real estate, anchoring was firstly shown by Northcraft and Neale (1987). The main factor influencing investors in Kenya is Anchoring bias (Waweru et al., 2014). In consequence of the discussion, a hypothesis is generated as:

**H2:** Anchoring bias has a significant positive effect on investment decisions.

### Risk Perception and Decision Making

Risk can be minimized by understanding the investor’s perception (Odean, 1998). However, traditionally, investors’ decisions are based on irrational behavior owing to different biases. For example, individual investors often focus more on potential negative outcomes than positive ones. Behavioral finance uses perceptions from other science and business areas to analyze investors’ choices. Some human behavior studies consider how investors make financing decisions, while neurologists have researched how investors’ mindsets can affect their financial decisions (Akhtar et al., 2020).

Moreover, behavior examined how investors make and act upon their decisions. Risk perception appears to be reflected in subjective behaviors influenced by other factors (Weber et al., 2004). Thus, Risk perception plays a fundamental role in investors’ behavior. When an individual makes a judgment about a financial instrument, the judgment process involves behavioral risk indicators and financial risk measures. In addition, Risk perception affects behavioral finance.

Moreover, Forlani and Mullins (2000) examined perceived risk and found significant links with behavioral biases. In previous researches, Risk perception is used as an intervening variable. Houghton et al. (2000) also investigated that Risk perception mediated the relationship between cognitive biases and the decision to start a venture. They also proposed that other biases need to be considered for future research. Nguyen and Rozsa (2019) investigated that Risk perception and risk tolerance significantly affect investment decision-making.

Investment involves the allocation of money with the hope of gaining returns and benefits in the future. These returns are based on whether the investor’s behavior is rational or irrational and are associated with risk. Investors face difficulties regarding how much they can invest in the stock market. Traditional finance clearly explains that investors should not make decisions based on emotion and behaviors. Traditional finance accepts that an investor is a rational person who can fairly process all details and investors are expected to be more rational and risk aversion.

**H3:** Risk perception mediates the effect of Optimism bias on investment decisions.

**H4:** Risk perception mediates the effect of Anchoring bias on investment decisions.

### Moderating Role of Information Asymmetry

In most manifestations in real estate market, asymmetric information is the norm (Lützkendorf and Speer, 2005). For more than two decades, the investigation of asymmetric information has been the salient economic theory. Asymmetric information is a very common feature of market interaction (Bukhari et al., 2021). When one party inside the market approaches more information than the other party, Information asymmetry occurs (Lützkendorf and Speer, 2005). In the context of asymmetric information, one party convinces the other party upon the quality and price of any product. The previous literature investigated asymmetric information has been conducted between principal and agent as principal is investor and manager is an agent. Literature has also established that Information asymmetry affects real investment in various ways and it gives satisfaction to investors (Rao et al., 2021).

Harris and Raviv (2010) also examined why managers (one party) had taken the informational advantage over shareholders (opposite part). To take the exclusive benefits, they retain the information that helps them pursue favorable investment decisions (1991). Property related data is not accessible for forthcoming the buyer’s/tenant’s informational asymmetries. Hence information asymmetries arise in real estate markets, as in other industries. The party offering a service or product (the agent) holds or can process information better than the party who requires it (Stiglitz, 2000). The decision-makers need a certain amount of financial decision-making information (Chewning and Harrell, 1990).

An investigation has been made in prior studies related to providing quality information and accurate data for investment decision-making. Ling et al. (2016) stated that the lack of supply of quality data on the commercial level derives the extremity of real estate unpredictability.

**H5a:** Information asymmetry moderates the effect of Optimism bias on investment decisions.

**H5b:** Information asymmetry moderates the effect of Anchoring bias on investment decisions.

An empirical study investigated that information plays a crucial role in financial institutions’ planning and decision-making. The information goes beyond subject domain boundaries and shows awareness of psycho-social barriers in acquisition and handling. Another study also investigated the importance of information in financial and strategic decision making and concluded that complete information leads to better decision making (Citroen, 2011). Information symmetry leads to the inauspicious selection of alternatives and market failure (Ahmad et al., 2021). These both are major effects of Information asymmetry. Adverse selection is a term utilized in financial aspects that alludes to a cycle wherein undesired outcomes happen when purchasers and dealers approach unique/insufficient data (Cohen and Siegelman, 2010; Boone, 2020). Market failure is when an individual will face challenges because the cost that could acquire will not be felt by the gathering facing the challenge (Jackson and Jabbie, 2019). The other consequence of asymmetric information is the agency problem between principal and agent. This phenomenon is also called the agency dilemma (Ross, 1973; Chod and Lyandres, 2020). The agency problem is created when there is a conflict between an agent and principal (Liao et al., 2009). One of the reasons for this conflict is asymmetric information. Empirical research investigated that the agency problem disturbs the self-interest of both parties (Chod and Lyandres, 2020).

**H6:** Information asymmetry moderates the effect of Risk perception on investment decision making.

**FIGURE 1:**
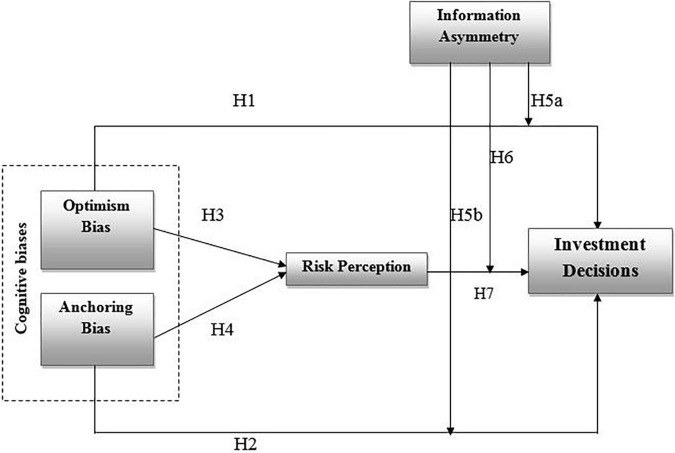
Conceptual framework.

In the above Figure 1, optimism and Anchoring bias are the predictors, which are effecting to investment decisions *via* the Risk perception (mediator). Information asymmetry works as a moderating variable between Risk perception and decision making.

## Methods

The methodological part of this exploration is to measure the effect of cognitive biases on investment decision-making and explore the mediating role of Risk perception. Moreover, the methodology part also examines the moderating effect of Information asymmetry.

### Target Population and Sample Size

One of the major and central aims of the research was to put attention on real investors. For this purpose, the target population of this study was the investors of the real estate sector. The study comprises only primary data, which will be assembled by utilizing the survey through the questionnaire. In the present research, the questionnaire included 34 items for measuring the variables. According to Hair et al. (2017) recommendations, the admissible sample size is the ratio between 5 and 10 observations per estimable criterion. According to Hair et al. (2017), it has been explained that the more admissible sample size should have the 10:1 ratio. Here, the lower limit is selected for creating the number of respondents.

### Data Collection

A pilot study has been conducted for translation of the questionnaire. The pilot study aims to evaluate the feasibility of the questionnaire on a trial basis (Thabane et al., 2010). During a pilot study, the instrument is given to 30 literate financial investors. These 30 respondents have confirmed the instrument’s content validity and face validity. In the prescribed description of the given sample (Table 1), data collection is done using a structured questionnaire sent to 340 respondents, 13 questionnaire are discarded due to incomplete information, 10 questionnaires are not received so the final 317 are accurate and complete. Therefore, the sample size is 317 for the analysis. The net response rate is 93.2%. A convenient sampling method is used to select respondents to provide the highest degree of responses.

**TABLE 1:** Break down of sample size.

	Composition of questionnaire	
Particulars	Number of questionnaires distributed	Percentage (%)
Questionnaires distributed	340	100
Questionnaire completed	317	93.2
Questionnaire discarded	13	3.82
Questionnaire not received	10	2.94

### Measurement

There are two cognitive variables consists of Optimism bias and Anchoring bias. Optimism bias has nine items adopted from Mishra et al. (2008) (“In uncertain times, I usually expect the best” is a sample item). Anchoring bias has two items adopted from Waweru et al. (2008). The sample item is “My investment is affected by my recent investment experiences.” The Risk perception scale was measured using the four items proposed by Weber et al. (2004) and a sample item is “I invest 10% of my annual income in a moderate growth mutual fund.” The Information asymmetry has eight items adopted from Mahaney and Lederer (2011), “My investment agent describes all the issues to me openly” is a sample item. The dependent variable was investment decision making consisting of ten items adopted from Mayfield et al. (2008) “I decide to invest every year” is a sample item of decision making.

### Reliability Analysis

Cronbach’s alpha is a measurable action of internal equilibrium and consistency known as scale reliability. It is also very useful to determine whether the scale we are using is fit for purpose or not (Taber, 2018). The minimum permissible value for Cronbach’s alpha is 0.70 (Taber, 2018), and if the outcome value approaches below the accepted value, internal consistency is low. The results indicate that Cronbach’s alpha value of all the factors is greater than 0.70 (Table 2).

**TABLE 2:** Reliability statistics.

	Cronbach’s alpha	Number of items
Investment decision making	0.870	10
Anchoring bias	0.720	2
Optimism bias	0.765	9
Risk perception	0.823	5
Information asymmetry	0.768	8

### Demographic Variables

Table 3, the frequency of age is given below as there are 4.2% respondents with the age of less than 20 years, 5.7% respondents having age of 21–30 years, 27.4% respondents lies between 31 and 40 years, 42.9% respondents exist between 41 and 50 years, and 19.8% respondents having age of more than 50 years. It analyzes 41–50 years of investors in the real estate market.

**TABLE 3:** Descriptive statistics.

	Frequency	Percent
Age		
Less than 20	13	4.2
21–30	18	5.7
31–40	87	27.4
41–50	136	42.9
More than 50	63	19.8
Gender		
Female	44	14.2
Male	273	85.8
Education		
Matric	54	17.0
Intermediate	76	24.1
Bachelors	99	31.1
Masters	61	19.3
Post graduate	27	8.5
Financial experience		
Less than 1 year	45	14.2
1–5 years	69	21.7
6–10 years	165	51.9
More than 10 years	38	12.3

The frequency of gender is given below as there are 14.2% female respondents and 85.8% male respondents, which analyzes that males are more engaged in real estate investment the females. This analysis is consistent with another research in Sweden (Pauli et al., 2014) that males are more engaged in the real estate business than females.

The frequency of education is given below as there are 17.0% respondents having matriculation, 24.1% respondents are intermediate, 31.1% respondents with the education of bachelors, 19.3% respondents with the education of masters, and 8.5% respondents with the education of post-graduates. It analyzes that most investors in developing countries are bachelor degree holders and very few postgraduates.

The frequency of frequency is given below as there are 14.2% respondents with financial experience of less than 1 year, 21.7% respondents with financial experience of 1–5 years, 51.9% respondents with financial experience 6–10 years, and 12.3% respondents with the financial experience of more than 10 years.

### Statistical Technique

PROCESS is known as a calculating tool available for SPSS and SAS. Its major purpose is to clarify the execution of mediation, moderation, and conditional process accompanied by perceived variables (Hayes, 2017).

Table 4, the effect of Anchoring bias has been analyzed on the mediator who is Risk perception. The table indicates that the effect of Anchoring bias on Risk perception is positively significant. Consequently, Anchoring bias is a significant predictor of Risk perception, β = 0.687, SE = 0.0410, 95% CI, (0.7335–0.9571), *p* = 0.0000 which is *p* < 0.05 and Risk perception is the significant predictor of investment decision making, β = 0.7835, SE = 0.0791, 95% CI, (0.2737–0.5316), *p* = 0.0000 which is *p* < 0.05. These results support the mediation analysis. It represents the effect of independent variable Anchoring bias on investment decision-making without considering mediator Risk perception.

**TABLE 4:** Effect of cognitive biases (Anchoring bias and Optimism bias) on investment decision making (DM) *via* mediating role of Risk perception.

Y (output)	Mediator	Model	*B*	SE	*T*	*p*	LLCI-ULCI
Investment decision making (DM)	Risk perception (RP)	Anch → RP (path a)	0.6867	0.0410	16.7837	0.000	0.7335–0.9571
		RP → DM (path b)	0.7835	0.0791	9.9008	0.000	0.2737–0.5316
		Anch → DM (path c)	0.6670	0.0717	9.3036	0.000	0.8276–1.0551
		Anch → DM (path c′)	0.4621	0.0565	8.1788	0.000	0.4498–0.7522
		Indirect effect of RP	0.5387	0.738			0.4045–0.6937
		Opt → RP (path a)	0.8453	0.0567	14.9067	0.000	0.7335–0.9571
		RP → DM (path b)	0.4026	0.0654	6.1550	0.000	0.2737–0.5316
		Opt → DM (path c)	0.9414	0.0577	16.3199	0.000	0.8276–1.0551
		Opt → DM (path c′)	0.6010	0.0767	7.8385	0.000	0.4498–0.7522
		Indirect effect of RP	0.3403	0.0693			0.2079–0.4777

Anchoring bias is a significant predictor of investment decision making after controlling the mediator Risk perception, β = 0.4621, SE = 0.0565, 95% CI, (0.4498–0.7522), *p* = 0.0000 which is *p* < 0.05. The effect of Optimism bias has been analyzed on the mediator who is Risk perception. The table indicates that the effect of Optimism bias on Risk perception is positively significant and Optimism bias is a significant predictor of Risk perception, β = 0.8453, SE = 0.0567, 95% CI, (0.7335–0.9571), *p* = 0.0000 which is *p* < 0.05 and Risk perception is the significant predictor of investment decision making, β = 0.4026, SE = 0.0654, 95% CI, (0.2737–0.5316), *p* = 0.0000 which is *p* < 0.05. These results support the mediation analysis. The above table represents the effect of independent variable Optimism bias on investment decision making without the mediator Risk perception. Optimism bias is a significant predictor of investment decision making *via* Risk perception, β = 0.9414, SE = 0.0577, 95% CI, (0.8276–1.0551), *p* = 0.0000 which is *p* < 0.05. Moreover, results indicated that there is a partial mediation exist against the effect of cognitive biases on decision making *via* mediating role of Risk perception as all paths are significant.

### Regression Analysis

Specifically, regression analysis described how dependent value Y fluctuates when any independent variable decreases or increases by holding the independent variable constant. The highest value of R is desirable (Cohen et al., 2013).

The below (Table 5) indicates a 54.2% direct association between Anchoring bias and decision making. Optimism bias and decision making are 75.2% associated. Anchoring bias is positively associated with the mediator called Risk perception, which is 75.9%. The mediator Risk perception is positively 72.3% associated with investment decision making. The relationship of Anchoring bias through Risk perception with investment decision making is 72.3% and the association of Optimism bias through Risk perception with investment decision making is 79.6%. The value of *R*^2^ indicates the proportion of variance in the dependent variable predicted from the independent variable. The sig values of the coefficients indicate whether these relationships are statistically significant. The significant value, denoted as sig in the above table, indicates the acceptance of the entire hypothesis. A value less than 0.05 (95% CI) indicates a significant relationship between concerned variables in the selected model. The above table indicates that the entire hypothesis shows 0.000 values, which means the relationship is statistically significant.

**TABLE 5:** Model summary.

Model	*R*	*R* ^2^	Sig	Unstandardized coefficients
Anch → DM	0.542	0.294	0.000	0.524
Opt → DM	0.752	0.566	0.000	0.942
Anch → RP	0.759	0.576	0.000	0.688
Opt → RP	0.721	0.520	0.000	0.845
RP → DM	0.723	0.523	0.000	0.773
Anch + RP → DM	0.723	0.522	0.000	−0.013/0.783
Opt + RP → DM	0.796	0.633	0.000	0.601/0.403

### Mediated Moderation Effect of Information Asymmetry

In this research, it has been proposed that Information asymmetry moderates the effect of Anchoring biases on investment decision-making and moderates the effect of mediator, Risk perception on investment decision making. The moderation effect has been tested with the (Model 15) of PROCESS macro (Preacher and Hayes, 2008; Hayes, 2017). In the given Table 6, it has been indicated that the moderator Information asymmetry significantly moderates the direct effect of Anchoring bias on investment decision making. The effect of Anchoring bias on investment decision making is significantly moderated by Information asymmetry (coeff = 0.1718, BootSE = 0.1112, 95% CI, 0.3919–0.0474). The effect of Anchoring bias on Risk perception has a significant effect. The effect of Risk perception on investment decision making is also moderated by Information asymmetry (coeff = 0.1265, BootSE = 0.1169, 95% CI, 0.1039–0.3570).

**TABLE 6:** Mediated moderation effect of Information asymmetry.

	Y (output)		Bootstrap 95% CI				
X	Mediator (M)	Moderated (W)	Int-term	Coeff	Boot SE	LLCI	ULCI
Anch	Risk perception	Information asymmetry	X × W	0.1718	0.1112	0.3910	0.0474
			M × W	0.1265	0.1169	0.1039	0.3570
Opt			X × W	0.0576	0.1149	0.1054	0.2847
			M × W	0.0896	0.0989	0.2842	0.1690

The second cognitive bias in this research is Optimism bias. The effect of Optimism bias on investment decision making is also significantly moderated by Information asymmetry (coeff = 0.0576, BootSE = 0.1149, 95% CI, 0.1054–0.2847). The effect Risk perception on investment decision making is significantly moderated by the moderator Information asymmetry (coeff = 0.0896, BootSE = 0.0989, 95% CI, 0.2842–0.1690). Hayes and Scharkow (2013) also reported a method for evaluating mediation moderation by an index value, BootLLCI, and BootULCI. The zero value did not fall within the upper or lower limit of the class interval and the index value is also less than 5%. This indicates that the model is perfectly mediation moderation (Table 7).

**TABLE 7:** Index of mediation moderation.

Index	Boot SE	LL (95% CI)	UP (95% CI)
0.038	0.03	0.003	0.14

*N = 317, B = unstandardized regression coefficient. Bootstrap sample size = 5,000. Confidence interval = 95%.*

*LL, lower limit; UL, upper limit; SE, standard error.*

### Two-Way Interaction Effect

The management research is fully crowded with the theories that describe that the effect of a causal variable on outcome variable depends on one another variable called moderator (Dawson, 2014).

**FIGURE 2:**
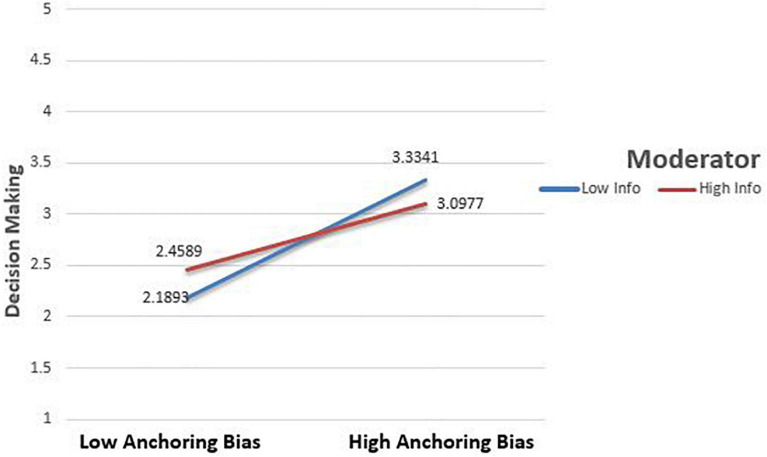
Interaction effect between Anchoring and Decision Making.

The above Figure 2 demonstrates that Anchoring bias and investment decision making is always positive. Still, it is far more for low Information asymmetry and far low when Information asymmetry is high. If there is high Information asymmetry, then the slope is less steep, which depicts that the moderator changes the association of Anchoring bias and decision making. Consequently, Information asymmetry reduces the effect of Anchoring bias on investment decision making.

**FIGURE 3:**
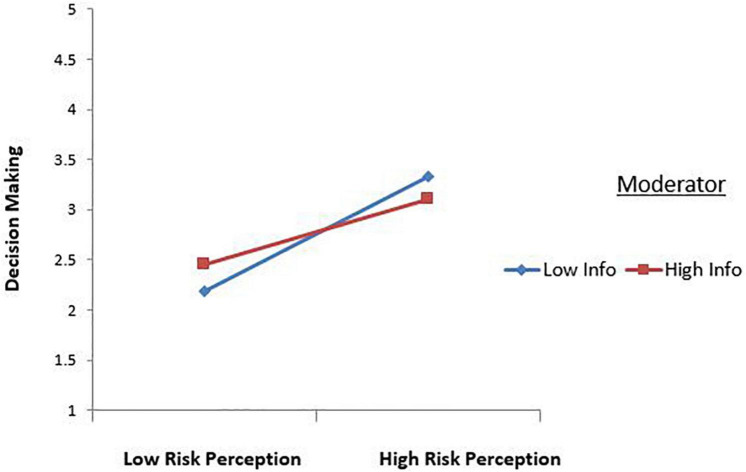
Interaction effect between Risk Perception and Decision Making.

The above Figure 3 demonstrates that Risk perception and investment decision-making are always positive. Still, it is far more for when there is low Information asymmetry and far low when Information asymmetry is high. If there is high Information asymmetry, then the slope is less steep, indicating that the moderator changes the association of Risk perception and decision-making. Consequently, Information asymmetry reduces the effect of Risk perception on investment decision making.

**FIGURE 4:**
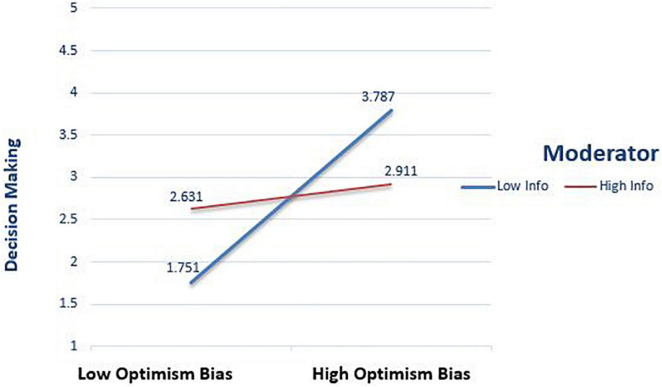
Interaction effect between Optimism Bias and Decision Making.

The above Figure 4 demonstrates Optimism bias and investment decision making is always positive but far more for when there is low Information asymmetry and far low when Information asymmetry is high. If there is high Information asymmetry, then the slope is less steep, which depicts that the moderator changes the association of Optimism bias and decision making.

## Discussion

This study investigates the decision-making perception of real estate investors. In behavioral finance, investment decisions are complicated activities for the investors. Generally, investors face many complex financial situations of uncertainty when making decisions. This uncertainty affects investors’ perception and leads to the worst decision making. Still, in this highly stimulating property market industry, it is necessary to take advantage of all opportunities and employ the resources of information. In this complex situation, cognitive biases affect investors’ decisions and revamp their performance of investors.

The Anchoring bias has a significant positive effect on investment (Figure 2). This finding is similar to Simmons’s (1999) and Evans (2002) findings. They investigated that investors utilize current prices before making investment decisions and presume that they are accurate. Investors use previous prices to make investment decisions (Waweru et al., 2014). Optimism bias has a positive effect on investment decisions (Figure 3). This finding is consistent with the study examining that investor’s decisions are significantly influenced by Optimism bias (Riaz and Iqbal, 2015; Jehiel, 2018; Limongi Concetto and Ravazzolo, 2019).

Risk perception mediates the effect of cognitive biases on investment decisions. The finding is similar to Greene (1998) and Cassar and Friedman (2009), who found that Risk perception affects investment decisions. Information asymmetry is a problem of prejudiced information distribution between agent and principal to take discriminatory advantage. This inequitable distribution of information enhances uncertainty (Newell and MacFarlane, 2006), ultimately leading to imprecise investment decisions. Information asymmetry significantly moderates the effect of cognitive biases on investment decisions (Chewning and Harrell, 1990). The study’s findings confirm that asymmetrical information negatively influences investment decision making (Gallimore et al., 2000) because investors need some sort of relatable information before investing (Chewning and Harrell, 1990) (Figure 4).

The results of the study suggested that the investment strategy relying on fast and frugal rules that would give better returns to investors. Based on our findings, researchers would like to suggest that investors should not rely on market information as Fama (1970) reported, but conduct a proper analysis on the behavior of the investors, develop quantitative investment measures and establish investment objectives and constraints.

### Conclusion and Implications

This study aimed to investigate the effects of cognitive biases on decision-making and explore the mediating effect of Risk perception and the moderating role of Information asymmetry on the effect of cognitive biases on investment decisions.

The results explored that cognitive biases positively affect investment decision making. In this study, two major cognitive biases are taken, i.e., Anchoring bias and Optimism bias positively influence decision making. This study indicates that Risk perception mediates cognitive bias on investment decision-making in the investment industry. It means that if an investor has strong beliefs in his ability to cope with situations when making investment decisions, cognitive biases positively affect decision-making.

In this study, we combine the theoretical fields of cognitive psychology and perception of risk along with the moderating effect of Information asymmetry on investment decision. Thus, the study makes an academic and practical implications by providing further insights into the cognitive biases and decision-making *via* the mediating role of Risk perception and the moderating effect of Information asymmetry. This study would be academically contribute in the curriculum of business studies especially the new horizons for the philosophy degree and also the behaviors of the investors. This study provides several practical implications for the government and real estate investors. For instance, the government should conduct seminars and workshops on the financial securities knowledge and behavior for real estate investors to reduce biases in their investment decisions, thereby helping the real estate market prosper. In the real estate context, awareness should be made for the brokerage houses and investors regarding the cognitive biases and as well as Risk perception.

This study contributes to the existing literature on cognitive biases and investment decision making. It is also helpful in the educational sector for business management studies, i.e., MBA students. This research will also provide insights into how situational (Anchoring) and emotional bias (Optimism) work together. This study enhances the skill set of financial advisors and investors themselves by a better understanding of investor’s goals. Furthermore, investors’ decisions are very important to set the market trends and enhance the economy. The real estate market is risky, but it is an investment-intensive sector that affects the economy and enhances other auxiliary activities such as mortgage enterprises. Therefore it is necessary to make rational and efficient decisions at investment. This study’s major role is to inform the investors about the hazards of Information asymmetry when making decisions. Generally, investors are unconscious of biases and thorough financial theories.

Hence investors behave irrationally by taking into account all prior information called Anchoring bias and optimism to maximize their profits or returns. All investment decisions are based on accurate information and past data that is perfect for decision making. In behavioral finance, biases are an individual’s judgments about specific things that they like and dislike, with such thoughts varying from person to person. Some factors that influence investors’ choices and behavior exist in investors’ thoughts, beliefs, and perceptions as biases. Consequently, this study helps the investors enforce the biases accurately and enhance the business sector. Moreover, this study helps to reduce the problem of asymmetrical information and enhances the investor’s efficiency. This exploration can be an important expansion to the literature in behavioral finance.

### Limitations and Future Recommendations

This study explores the impact of major cognitive biases on investment decisions in the specific context of developing countries which manifests that the sample size is limited. Future research should be at a huge geographical level for more generalized results. In this study, only investors are taken as population, but stock exchange investors should be taken as a unit of analysis in future research. In addition, the study can be done on the investors of the commodity market. The findings of this study is limited to the moderator, but in future, research can be done by investigating the moderating role of financial literacy (Nauman Sadiq and Asad Azad Khan, 2019; Sabir et al., 2019), Gender and Personality Traits (Gul, 1984) and also personality types. The study can also be done to the emotional intelligence of investors as an independent variable. This study can explore if other cognitive biases (i.e., overconfidence bias, herding bias, availability bias, and representative bias) are taken into the research. This study will explore more if it conducts on long-term and short-term investment intentions. Furthermore, this study used the convenience sampling method, but other sampling techniques can be used in future.

## Data Availability Statement

The raw data supporting the conclusions of this article will be made available by the authors, without undue reservation.

## Ethics Statement

The studies involving human participants were reviewed and approved by the Riphah International University, Management Sciences Department. The patients/participants provided their written informed consent to participate in this study.

## Author Contributions

MZ identified the problem area. MN did work on the contribution and conceptual framework. RF did work on the data collection. MI has done work on the contribution and limitation of the study. All authors listed have made a substantial, direct, and intellectual contribution to the work, and approved it for publication.

## Conflict of Interest

The authors declare that the research was conducted in the absence of any commercial or financial relationships that could be construed as a potential conflict of interest.

## Publisher’s Note

All claims expressed in this article are solely those of the authors and do not necessarily represent those of their affiliated organizations, or those of the publisher, the editors and the reviewers. Any product that may be evaluated in this article, or claim that may be made by its manufacturer, is not guaranteed or endorsed by the publisher.
